# Phenylpropanoid Derivatives from the Tuber of *Asparagus cochinchinensis* with Anti-Inflammatory Activities

**DOI:** 10.3390/molecules27227676

**Published:** 2022-11-08

**Authors:** Jingyi Yue, Nan Zhang, Tao Xu, Jutao Wang, Baixiang Cai, Yang Yu

**Affiliations:** 1 Wuhu Life and Health Engineering and Technology Research Center, Wuhu Institute of Technology, Wuhu 241006, China; 2 Department of Pharmacy, The Second Affiliated Hospital of Anhui University of Chinese Medicine, Hefei 230001, China; 3 Department of Biological and Pharmaceutical Engineering, West Anhui University, Luan 237012, China; 4 School of Pharmacy, Anhui University of Chinese Medicine, Hefei 230012, China; 5 Institute of Medicinal Chemistry, Anhui Academy of Chinese Medicine, Hefei 230012, China; 6 Anhui Province Key Laboratory of Research & Development of Chinese Medicine, Anhui Academy of Chinese Medicine, Hefei 230012, China; 7 State Key Laboratory of Phytochemistry and Plant Resources in West China, Kunming Institute of Botany, Chinese Academy of Sciences, Kunming 650201, China

**Keywords:** phenylpropanoid, *Asparagus cochinchinensis*, anti-inflammatory activity

## Abstract

Three undescribed phenylpropanoid derivatives, including two new bibenzyl constituents (**1**–**2**), one new stilbene constituent (**3**), together with five known compounds stilbostemin F (**4**), dihydropinosylvin (**5**), 2-(4-hydroxyphenyl)ethyl benzoate (**6**), 1-(4-hydroxybenzoyl)ethanone (**7**), and 4-hydroxy-3-prenylbenzoic acid (**8**), were isolated from the tuber of *Asparagus cochinchinensis*. The structures of **1**–**8** were elucidated according to UV, IR, HRMS, 1D and 2D-NMR methods together with the published literature. All of the isolated compounds were assessed for anti-inflammatory activity by acting on lipopolysaccharide (LPS)-induced RAW 264.7 macrophage cells in vitro. The results showed that compounds **2** and **5** were found to inhibit the production of nitric oxide (NO) with the IC_50_ value of 21.7 and 35.8 µM, respectively. In addition, further studies found that compound **2** demonstrated concentration-dependent suppression of the protein expression of iNOS and exerted anti-inflammatory activity via the NF-κB signalling pathway. The present data suggest that phenylpropanoid derivatives from the tuber of *A. cochinchinensis* might be used as a potential source of natural anti-inflammatory agents.

## 1. Introduction

Nitric oxide (NO) is a key signaling molecule and regulates various physiological functions in many tissues of the human body [1,2]. However, an overproduction of NO is associated with many inflammatory diseases [3]. Hence, the inhibition of excessive production of NO may have a therapeutic benefit in controlling inflammation and discovering new drugs for reducing inflammation using natural bioactive compounds plays an important role in research [4].

The genus of *Asparagus* has been used as a vegetable and as medicines due to its soothing flavor and wealth of health benefits [5,6,7]. *Asparagus cochinchinensis* is an important traditional Chinese herbal plant, and the use of its tuber is employed for treating cutaneous inflammation, aging, hyperlipidemia, cardiovascular disease, bacterial infection, diabetes, constipation, and throat pain [8,9]. Phytochemical studies have demonstrated that it contains flavonoids, phenolics, steroidal glycosides, alkaloids, and polysaccharide compounds [10,11,12,13].

In this research, as part of the ongoing search for new chemical and anti-inflammatory constituents from *A. cochinchinensis* [14], phytochemical and biological studies of the tuber of *A. cochinchinensis* were carried out to explore the anti-inflammatory ingredients.

**Figure 1:**
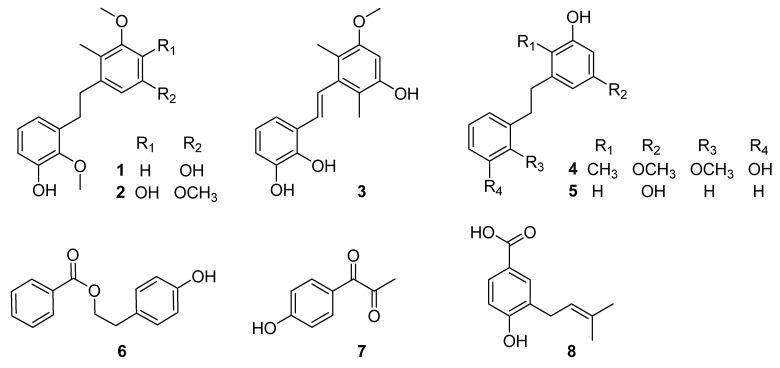
The chemical structures of compounds **1**–**8** obtained from the tuber of *A. cochinchinensis*.

Here, we describe the isolation, structure elucidation, and anti-inflammatory activity of a new compound, asparbiben A–C (**1**–**3**) –nd five known compounds stilbostemin F (**4**), dihydropinosylvin (**5**), 2-(4-hydroxyphenyl)ethyl benzoate (**6**), 1-(4-hydroxybenzoyl)ethanone (**7**), and 4-hydroxy-3-prenylbenzoic acid (**8**) (Figure 1).

## 2. Results and Discussion

### 2.1. Structure Elucidation

**Figure 2:**
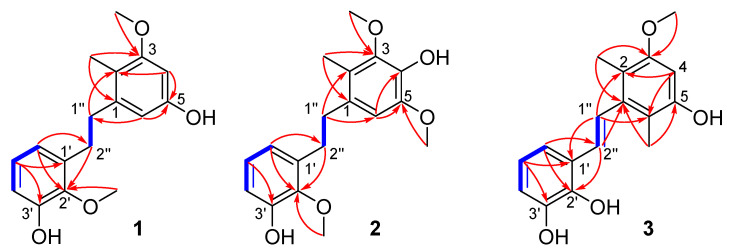
^1^H-^1^H COSY (**blue line**) and key HMBC (→) correlations of compound **1**–**3**.

**Figure 3:**
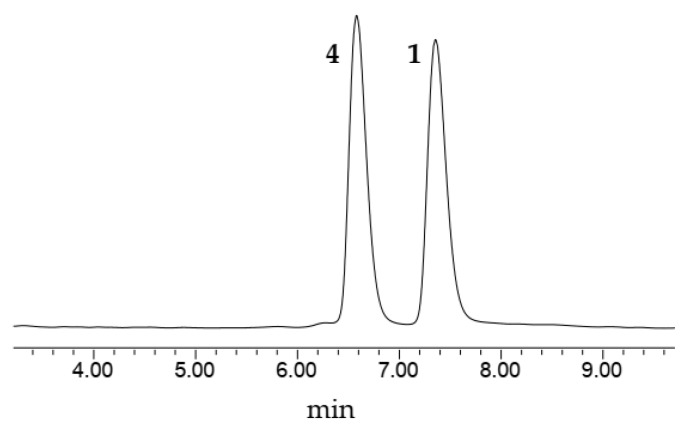
The HPLC analysis of compound **1** and **4**.

**Figure 4:**
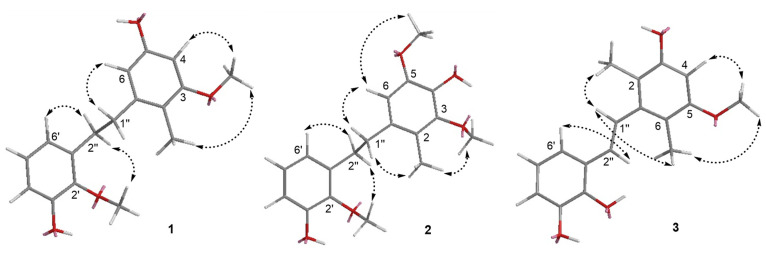
Key ROESY correlations (arrows) of compound **1**–**3**.

Compound **1** was purified as a light-yellow powder. The molecular formula was determined to be C_17_H_20_O_4_ according to the HR-ESI-MS analysis of *m/z* 287.1288 [M-H]^−^ (calculated value 287.1289 [M-H]^−^) (Figure S1.1), which was consistent with the 1D NMR spectroscopic data (Table 1). The UV spectrum displayed maxima absorption bands at *λ*_max_ 200 and 280 nm, whereas the IR spectrum showed hydroxy and aromatic ring functionalities at 3425 and 1607 cm^−1^, respectively (Figures S1.2 and S1.3). The ^13^C NMR and DEPT spectra (Figures S1.4–S1.9) displayed 17 carbon signals, including one methyl (*δ*_C_ 10.7), two methoxy (*δ*_C_ 55.8, 60.9), two methylenes (*δ*_C_ 32.5, 36.4), five methines, and seven olefinic non-protonated carbons. The ^1^H NMR and ^1^H-^1^H COSY spectrum (Table 1 and Figure 2) showed characteristic signals for one set of the ABC aromatic protons moiety at *δ*_H_ 6.62 (1H, dd, *J* = 7.9, 1.0 Hz), 6.68 (1H, dd, *J* = 7.9, 1.0 Hz), and 6.82 (1H, t, *J* = 7.9 Hz); two *meta* coupled aromatic protons at *δ*_H_ 6.22 (1H, d, *J* = 2.1 Hz), and 6.24 (1H, d, *J* = 2.1 Hz); one methyl doublet at *δ*_H_ 1.61 (3H, s); two additional methoxy groups (*δ*_H_ 3.73, 3H, s; *δ*_H_ 3.75, 3H, s); and two methine protons (*δ*_H_ 2.74, 2H, m; *δ*_H_ 2.74, 2H, m). The above data indicated that compound **1** was a bibenzyl compound [15] and resembled the known compound **4**. Nevertheless, they had different polarity in the HPLC analysis (Figure 3). The HMBC correlations from the methyl doublet 2-CH_3_ (*δ*_H_ 2.06) to C-3 (*δ*_C_ 160.0), together with 3-OCH_3_ (*δ*_H_ 3.73) to C-3 (*δ*_C_ 160.0), indicated that one methoxy group was linked at C-3 not at C-5 in compound **1**. The ROESY correlations between methyl protons (*δ*_H_ 1.61) and methoxy protons (*δ*_H_ 3.73, 3-OCH_3_) were consistent with the above speculation (Figure 4). Thus, the structure of compound **1** was established as 3,2′-dimethoxy-2-methyl-5,3′-dihydroxy-bibenzyl (Figure 1) and named asparbiben A.

Compound **2** was obtained as a pale-yellow powder. Its molecular formula was determined to be C_18_H_22_O_5_ by HRESIMS analysis (*m/z* 317.1392, calcd. for C_18_H_21_O_5_ [M-H]^−^, 317.1394) (Figure S2.1), corresponding to eight degrees of unsaturation. The 1D NMR data (Table 1) and UV absorption feature (Figures S2.2 and S2.3) of compound **2** showed high similarity with those of **1**, revealing that compound **2** was a structural congener of **1**. There were remarkable differences between the two sets of NMR data (Figures S2.4–S2.9), especially the presence of an additional methoxy group (*δ*_C_ 56.6), while the absence of the aromatic protons at C-4 (*δ*_C_ 138.5) in **2** compared to those of **1** indicated that the main difference was the substituent of C-4/5. Moreover, the C-4 of **2** might has been hydroxy-substituted. In the HMBC spectrum of **2** (Figure 2), the correlations from H-1″ (*δ*_H_ 2.77) to C-2 (*δ*_C_ 122.7), 2-CH_3_ (*δ*_H_ 2.14) to C-1 (*δ*_C_ 132.2) suggested that the methyl doublet was also linked at C-2. The hydroxy group was located at C-4 by the diagnostic HMBC correlation between H-6 (*δ*_H_ 6.44) and C-4 (*δ*_C_ 138.5). The pivotal HMBC correlation from H-6 to C-5 (*δ*_C_ 147.5), and 5-OCH_3_ (*δ*_H_ 3.74) to C-5 indicated another methoxy group connection at C-5. In addition, the ROESY correlations were consistent with the above speculation (Figure 4). Therefore, compound **2** was determined as 3,5,2′-trimethoxy-2-methyl-4,3′-dihydroxy-bibenzyl and named asparbiben B.

Compound **3** was isolated as brown powder, and its molecular formula was assigned as C_17_H_18_O_4_ based on its HRESIMS data, m/z 285.1133 [M-H]^−^ (calculated for C_17_H_17_O_4_, 285.1132) (Figure S3.1), indicating nine degrees of unsaturation. The IR spectrum showed hydroxy and aromatic ring functionalities at 3425 and 1590 cm^−1^ (Figures S3.2 and S3.3). The ^1^H-NMR (Table 1) (Figures S3.4–S3.9) spectrum of compound **3** also exhibited ABC aromatic protons moiety at *δ*_H_ 6.66 (1H, t, *J* = 7.7 Hz), 6.69 (1H, dd, *J* = 7.7, 1.9 Hz), 7.02 (1H, d, *J* = 7.7, 1.9 Hz); two methyl doublets at *δ*_H_ 2.09 (3H, s), 2.12 (3H, s); one methoxy group at *δ*_H_ 3.74 (3H, s); a double bond signal at *δ*_H_ 6.75 (1H, d, *J* = 16.7 Hz); and 7.05 (1H, d, *J* = 16.7 Hz) identified as *trans* by the coupling constant, together with one aromatic proton *δ*_H_ 6.36 (1H, s). The ^13^C-NMR and DEPT spectrum data (Table 1) showed two benzene ring carbons and one pair of olefin carbons *δ*_C_ 98.4, 114.8, 115.2, 116.2, 118.3, 120.4, 126.2, 128.3, 130.6, 140.9, 144.4, 146.4, 154.5, and 157.3; two methyl data *δ*_C_ 12.9 and 13.0; and one methoxy data *δ*_C_ 55.9. The 1D NMR (Table 1) spectra of **3** were similar to **1** and **2** except for the *trans* double bond, indicating the skeleton of **3** as a stilbene compound. The HMBC correlations (Figure 2) of the H-1″ (*δ*_H_ 7.05) with C-2 (*δ*_C_ 116.2) and C-6 (*δ*_C_ 115.2), and the two methyl doublets at *δ*_H_ 2.09 and 2.12 with C-1 (*δ*_C_ 140.9) assigned the locations of two methyl groups at C-2 and C-6, respectively. One methoxy group was linked to C-3, which was demonstrated by the following correlations in the HMBC spectrum: 2-CH_3_ and 3′-OCH_3_ to C-3 (*δ*_C_ 157.3). Moreover, the ROESY cross-peaks (Figure 2) between H-1″ (*δ*_H_ 7.05) and 2/6-CH_3_ (*δ*_H_ 2.09, 2.12), and between H-4 (*δ*_H_ 6.36)/2-CH_3_ and 3-OCH_3_ (*δ*_H_ 3.74) also supported these assignments. Consequently, **3** was concluded to be 3-methoxy-2,6-dimethyl-5,2′,3′-trihydroxy- stilbene and named asparbiben C.

The known compounds (**4**–**8**) were identified on the basis of a detailed spectroscopic interpretation in comparison to the reported values in the references, to be stilbostemin F (**4**) [16], dihydropinosylvin (**5**) [17], 2-(4-hydroxyphenyl)ethyl benzoate (**6**) [18], 1-(4-hydroxybenzoyl)ethanone (**7**) [19], and 4-hydroxy-3-prenylbenzoic acid (**8**) [20] (Figure 1).

### 2.2. Inhibitory Effects of Compounds **1**–**8** on NO Production of LPS-Activated RAW 264.7 Cells

An MTT assay was used to evaluate the cytotoxic effects of **1**–**8** on RAW 264.7 cells in vitro, with aminoguanidine hydrochloride (AH) used as a positive control. The results showed that none of the compounds or positive control exhibited significant cytotoxicity at a concentration of 50 µM (over 75% cell survival). Furthermore, the inhibitory effect of **1**–**8** on the production of NO in LPS-induced RAW 264.7 cells was measured by the Griess method [21]. The inhibitory effect of **1**–**8** on NO release is shown in Table 2. The results showed that two of the isolated compounds (**2** and **5**) displayed NO inhibitory activity (IC_50_ 21.7 and 35.8 µM) (positive control: Aminoguanidine hydrochloride, IC_50_ 18.4 ± 2.33 µM). Especially, by comparing compound **1**, **2** with **3**, we found when the methoxy group linked at C-4 could cause a dramatic promotion in the inhibitory activity. The NO inhibitory activity of compound **2** and **5** versus **3** suggested the bibenzyl compounds were more active than stilbene compounds and the Δ^1″(2″)^ double bond offers no assistance to anti-inflammatory activity. The above structure–activity relationship (SAR) was preliminary and needed to be validated.

### 2.3. Inhibitory Effects of New Compound **2** on LPS-Enhanced Inflammatory Mediators

**Figure 5:**
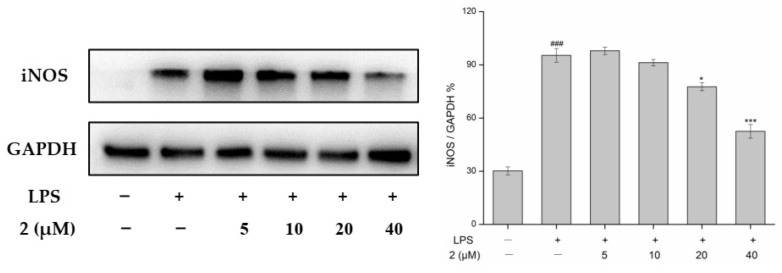
iNOS protein levels of compound **2** with different concentrations (5, 10, 20, 40 µM) were detected by a Western blot assay. (*** *p* < 0.001, * *p* < 0.05 compared to the LPS-treated group. ^###^ *p* < 0.001 compared to the blank group.).

Proinflammatory molecules, such as iNOS, are involved in inflammation-associated diseases and act as inflammatory mediators or activators of inflammatory pathways. Herein, Western blot analysis was performed to detect the protein expression of the inflammation markers iNOS as proteins of the NF-κB pathway [22]. Among all the isolated compounds, compound **2** displayed the strongest inhibition on NO release, so it was selected for further study. Protein expression levels were normalized against GAPDH. As shown in Figure 5, compound **2** treatment significantly inhibited LPS-induced expression of iNOS in RAW 264.7 cells. A down-regulation of iNOS expression in the presence of **2** at 40 µM was observed. In conclusion, these results suggested that **2** exerts anti-inflammatory activity, possibly via the NF-κB signaling pathway. However, further investigations are necessary to elucidate whether these compounds can act on other inflammatory mechanisms.

Previous research has shown that *A. cochinchinensis* is a potential therapeutic agent for inflammatory diseases [23,24]. iNOS was an important target for the NF-κB inflammasome pathway to prevent an inflammatory response [25]. Therefore, anti-inflammatory agent **2**, with some iNOS expression inhibitory activities, and the potential anti-inflammatory constituent **5** along with the benzofuranoid norlignans (asparlignan A and B) which were isolated from the aerial parts of *A. cochinchinensis* in the previous study [14] might form some of the effective ingredients for *A. cochinchinensis* to prevent inflammatory diseases.

## 3. Materials and Methods

### 3.1. General Experimental Procedures

The NMR spectra were performed on a Bruker AVANCE DRX-500 spectrometer, operating at 500 MHz for ^1^H, and 125 MHz for ^13^C (Bruker, Germany). The Fourier transform infrared (FTIR) spectra were recorded with KBr disks on a Bruker vertex-70 spectrometer (Bruker, Germany). The HRESIMS spectrum was obtained on a Shimadzu LC-TOFMS (Shimadzu, Japan). MPLC separation was performed on a Buchi sepacore (Buchi Labortechnik AG, Flawil, Switzerland) with a YMC gel ODS C_18_ column (45–60 μm, YMC Co., Ltd., Kyoto, Japan). Column chromatography (CC) was carried out on silica gel (200–300 mesh, Qingdao Marine Chemical Co., Ltd., Qingdao, China) and Sephadex LH-20 (GE Healthcare Bio-Sciences AB, Uppsala, Sweden). HSGF254 thin-layer plates were used (Qingdao Marine Chemical Co. Ltd., China). Preparative HPLC separation was conducted on an LC-3000 semi-preparation gradient HPLC system (Chuangxintongheng, Beijing, China) using a UV–vis detector and analysis with a RP-HPLC column (Shiseido CAPCELL PAK C_18_ column, 250 mm × 20 mm, 5 μm, Tokyo, Japan). CH_3_CN (HPLC grade) was obtained from CINC High Purity Solvents, Shanghai, China. Methanol, ethyl acetate were obtained from (AR) (Sinopharm Co., Ltd., Shanghai, China). Ultrapure water was obtained from a Milli-Q system (Milford, MA, USA).

### 3.2. Plant Materials

The tuber of *A. cochinchinensis* was collected in Lu’an, Anhui province, China. The plant material was identified by associate Prof. Tao Xu (West Anhui University) and a voucher specimen (TMD-10) has been deposited at the School of Pharmacy, Anhui University of Chinese Medicine.

### 3.3. Extraction and Isolation

The air-dried tubers of *A. cochinchinensis* (3 kg) were extracted three times with methanol to obtain a crude extract, then the extract was suspended in water, followed by extraction with EtOAc. The partial fraction from the EtOAc extract (12 g) was observed using a silica gel column (200–300 mesh) and eluted sequentially with CHCl_2_-CH_3_OH (100:0 to 0:1, *v*/*v*) to obtain four subfractions (Fr.A-Fr.E). Fr. B (570 mg) was separated with a Sephadex LH-20 column (CH_3_OH) and further purified with semipreparative HPLC (CH_3_CN-H_2_O, 60:40, *v*/*v*, 8 mL/min) to furnish compound **2** (10.0 mg, *t*_R_ = 30.2 min) and **7** (6.8 mg, *t*_R_ = 34.1 min). Fr. C (6 g) was put through silica gel column chromatography and eluted with CHCl_2_-acetone (15:1 to 5:1, *v*/*v*) to provide fractions C-1 to C-4. Meanwhile, the Fr.C-2 (2.1 g) was separated by MPLC using CH_3_OH-H_2_O (40:70–100:0, *v/v*, 8 mL/min), and followed by Sephadex LH-20 column (CH_3_OH) and further purified by pre-HPLC (CH_3_CN-H_2_O, 50:50, *v*/*v*, 8 mL/min) to give compound **4** (18.0 mg, *t*_R_ = 28.7 min) and **1** (8.0 mg, *t*_R_ = 33.7 min). Later, Fr.C-3 (1.5 g) was isolated by MPLC eluted with CH_3_OH-H_2_O (30:70–100:0, *v*/*v*), and purified with pre-HPLC (CH_3_CN-H_2_O, 50:50, *v*/*v*, 8 mL/min) to yield compound **5** (8.2 mg, *t*_R_ = 31.3 min) and **6** (13.1 mg, *t*_R_ = 40.1 min). Fr.D (4 g) was chromatographed through MPLC (CH_3_OH-H_2_O, 30:70–100:00, *v*/*v*, 8 mL/min) to give five subfractions Fr.D-1–Fr.D-5. Compound **8** (9.8 mg, *t*_R_ = 26.8 min) was isolated from Fr.D-4 (52 mg) through purification with semi-preparative HPLC (CH_3_CN/H_2_O, 30:70, *v*/*v*, 8 mL/min). Posteriorly, Fr.D-5 (86 mg) was further chromatographed on Sephadex LH-20 column using an isocratic solvent system of CH_3_OH and applied to pre-TLC (petroleum ether: acetone 2:1, *v*/*v*) to provide compound **3** (7.6 mg).

Asparbiben A (**1**): yellowish solid; UV (MeCN) *λ*_max_ 200, 280 nm; IR (KBr) *ν*_max_ 3425, 2938, 1607, 1597, 1471, 1305, 1194, 1147, 1104, 987, 751 cm^−1^; ^1^H and ^13^C NMR data (Table 1); HRESIMS *m/z* 287.1288 [M-H]^−^ (calcd for C_17_H_19_O_4_, 287.1289).

Asparbiben B (**2**): yellowish solid; UV (MeCN) *λ*_max_ 201, 280 nm; IR (KBr) *ν*_max_ 3495, 3340, 2942, 1616, 1589, 1497, 1471, 1321, 1289, 1199, 1143, 1050, 1000, 818, 762 cm^−1^; ^1^H and ^13^C NMR data (Table 1); HRESIMS *m/z* 317.1392 [M-H]^−^ (calcd for C_18_H_21_O_5_, 317.1394).

Asparbiben C (**3**): brown solid; UV (MeCN) *λ*_max_ 193, 214, 268 nm; IR (KBr) *ν*_max_ 3425, 2938, 1590, 1472, 1325, 1277, 1193, 1122, 1084, 977, 829, 729 cm^−1^; ^1^H and ^13^C NMR data (Table 1); HRESIMS *m/z* 285.1133 [M-H]^−^ (calcd for C_17_H_17_O_4_, 285.1132).

### 3.4. Cell Culture and NO Production Measurements

The experimental procedures were followed as per the literature [26]. Cell viability was evaluated using the MTT assay (5 mg/mL). The RAW264.7 cells were seeded into 96-well plates at density of 50,000 cells/well for 24 h. Then the cells were pretreated with the tested compounds for 30 min at 37 °C, and then stimulated with LPS (100 ng/mL) for 24 h. The Griess reaction was used to detect the NO level. Momentarily, the cell culture supernatant (50 μL) and Griess reagent (50 μL) were mixed with an equal volume for 10 min, and then the absorbance was monitored at 540 nm using a microplate reader. All the tested compounds were prepared as stock solutions with a concentration of 10 mM in DMSO. Aminoguanidine hydrochloride was used as the positive control group.

### 3.5. Western Blot Analysis

Cells (5 × 10^5^/well) were initially treated with different concentrations (5, 10, 20, 40 μM) of compound **2** and LPS (1 μg/mL) stimulation (Figure 5). Then the total proteins were extracted and immunoblotted as previously described [27,28]. Briefly, the RAW264.7 cells were lysed with 1% RIPA (radio-immunoprecipitation assay) (Amresco, Solon, OH, USA) to achieve the cellular lysates. The total proteins of the cellular lysates were measured by the BCA protein assay kit. Total proteins were separated by SDS-PAGE and transferred onto a PVDF membrane (Bio-Rad Laboratories, Hercules, CA, USA). Then the membranes were washed with TBST buffer, blocking with 5% non-fat milk for 2 h at 25 °C, and then incubated with primary antibodies for 12 h at 4 °C. After being washed with TBST buffer, the membranes were treated with a secondary antibody at room temperature and the protein bands were detected.

## 4. Conclusions

In summary, three undescribed phenylpropanoid derivatives along with five known compounds were co-isolated from the tuber of *A. cochinchinensis*. Among them, asparbiben A–C (**1**–**3**) were identified as new bibenzyl and stilbene derivatives. In addition, these isolated compounds enrich the chemical entities of naturally occurring phenylpropanoids and the structural diversity of the Asparagus family. The phenylpropanoid constituents, especially the bibenzyl derivatives, may act as potential anti-inflammatory agents and this has attracted the attention of many researchers [29,30]. In the bioassays, all the isolated compounds were screened for anti-inflammatory effects. The screened results indicated that compounds **2** and **5** exhibited a potential inhibitory effect on NO production, with an IC_50_ value of 21.7 and 35.8 µM, respectively. These results demonstrate that structurally different phenylpropanoid compounds in *A. cochinchinensis* may contribute its anti-inflammatory function. Importantly, the potential compound **2** decreased the protein expression levels of iNOS, indicating that **2** may be mediated via the suppression of an LPS-induced NF-κB inflammasome pathway. Taken together, phenylpropanoid derivatives are believed to be the main anti-inflammatory constituents of *A. cochinchinensis*. The present study lays the foundation for research into the potential therapeutic value of phenylpropanoid derivatives for inflammatory diseases.

## Figures and Tables

**Table 1:** ^1^H NMR (500 MHz) and ^13^C NMR (125 MHz) spectroscopic data of **1**–**3** in CD_3_OD (*δ* in ppm, *J* in Hz).

Position	1		2		3	
	*δ*_H_ (*J* in Hz)	*δ*_C_	*δ*_H_ (*J* in Hz)	*δ*_C_	*δ*_H_ (*J* in Hz)	*δ*_C_
1	-	143.2, C	-	132.2, C	-	140.9, C
2	-	116.1, C	-	122.7, C	-	116.2, C
3	-	160.0, C	-	147.4, C	-	157.3, C
4	6.24 (d, 2.1)	97.6, CH	-	138.5, C	6.36 (s)	98.4, CH
5	-	156.7, C	-	147.5, C	-	154.5, C
6	6.22 (d, 2.1)	108.9, CH	6.44 (s)	109.8, CH	-	115.2, C
1″	2.74 (m)	36.4, CH_2_	2.77 (m)	35.8, CH_2_	7.05 (d, 16.7)	128.3, CH
2″	2.74 (m)	32.5, CH_2_	2.77 (m)	32.5, CH_2_	6.75 (d, 16.7)	130.7, CH
1′	-	136.8, C	-	136.7, C	-	126.2, C
2′	-	147.2, C	-	147.3, C	-	144.4, C
3′	-	151.2, C	-	151.2, C	-	146.4, C
4′	6.68 (dd, 7.9, 1.0)	115.6, CH	6.68 (dd, 8.0, 1.5)	115.5, CH	6.69 (dd, 7.7, 1.9)	114.8, CH
5′	6.82 (t, 7.9)	125.2, CH	6.81 (t, 8.0)	125.0, CH	6.66 (t, 7.7)	120.4, CH
6′	6.62 (dd, 7.9, 1.0)	121.9, CH	6.59 (dd, 8.0, 1.5)	122.1, CH	7.02 (dd, 7.7, 1.9)	118.3, CH
2-CH_3_	2.06 (s)	10.7, CH_3_	2.14 (s)	11.3, CH_3_	2.09 (s)	12.9, CH_3_
3-OCH_3_	3.73 (s)	55.8, CH_3_	3.72 (s)	60.4, CH_3_	3.74 (s)	55.9, CH_3_
5-OCH_3_	-	-	3.74 (s)	56.6, CH_3_	-	-
6-CH_3_	-	-	-	-	2.12 (s)	13.0, CH_3_
2′-OCH_3_	3.75 (s)	60.9, CH_3_	3.72 (s)	60.8, CH_3_	-	-

**Table 2:** IC_50_ values of isolated compounds **1**–**8** inhibiting NO production in RAW 246.7 cells.

Compounds	IC_50_ (µM)
**1**	>50
**2**	21.7 ± 1.62
**3**	>50
**4**	>50
**5**	35.8 ± 2.01
**6**	>50
**7**	>50
**8**	>50
AH ^a^	18.4 ± 2.33

a: AH = Aminoguanidine hydrochloride was used as the positive control.

## Data Availability

Not applicable.

## Supplementary Materials

The following are available online at https://www.mdpi.com/article/10.3390/molecules27227676/s1, Figure S1.1. (-)-HRESIMS of compound 1. Figure S1.2. UV of compound 1. Figure S1.3. IR of compound 1. Figures S1.4–S1.9. NMR spectra of compound 1. Figure S2.1. (-)-HRESIMS of compound 2. Figure S2.2. UV of compound 2. Figure S2.3. IR of compound 2. Figures S2.4–S2.9. NMR spectra of compound 2. Figure S3.1. (-)-HRESIMS of compound 3. Figure S3.2. UV of compound 3. Figure S3.3. IR of compound 3. Figures S3.4–S3.9. NMR spectra of compound **3**.
