# Supplementary material for: Phenylpropanoid Derivatives from the Tuber of Asparagus cochinchinensis with Anti-Inflammatory Activities
Source: Molecules. 2022 Nov 8;27(22):7676. doi: 10.3390/molecules27227676 (PMC9697020; doi:10.3390/molecules27227676)
Supplement: Supplementary file 1 [file molecules-27-07676-s001.zip › molecules-2007659-supplementary.pdf]

Supplementary information

Phenylpropanoid Derivatives from the Tuber of *Asparagus cochinchinensis* with Anti-Inflammatory Activities

Jingyi Yue [1], Nan Zhang [2], Tao Xu [3], Jutao Wang [4, 5, 6], Baixiang Cai [3, 7, *], and Yang Yu [4, 5, 7, *]

[1] Wuhu Institute of Technology, Wuhu 241006, China
[2] Department of Pharmacy, The Second Affiliated Hospital of Anhui University of Chinese Medicine, Hefei 230001, China
[3] Department of Biological and Pharmaceutical Engineering, West Anhui University, Luan 237012, China
[4] School of Pharmacy, Anhui University of Chinese Medicine, Hefei 230012, China
[5] Institute of Medicinal Chemistry, Anhui Academy of Chinese Medicine, Hefei 230012, China
[6] Anhui Province Key Laboratory of Research & Development of Chinese Medicine, Hefei 230012, China
[7] State Key Laboratory of Phytochemistry and Plant Resources in West China, Kunming Institute of Botany, Chinese Academy of Sciences, Kunming 650201, China
* Correspondence: caibx103@126.com or caibaixiang@mail.kib.ac.cn (B.X. C.); yyang71@ahtcm.edu.cn or yuyang1@mail.kib.ac.cn (Y.Y.)

Contents of supplemental material

Figure S1.1. (-)-HRESIMS of compound **1**.

Figure S1.2. UV of compound **1**.

Figure S1.3. IR of compound **1**.

Figure S1.4-1.9. NMR spectra of compound **1**.

Figure S2.1. (-)-HRESIMS of compound **2**.

Figure S2.2. UV of compound **2**.

Figure S2.3. IR of compound **2**.

Figure S2.4-2.9. NMR spectra of compound **2**.

Figure S3.1. (-)-HRESIMS of compound **3**.

Figure S3.2. UV of compound **3**.

Figure S3.3. IR of compound **3**.

Figure S3.4-3.9. NMR spectra of compound **3**.

Error Margin (ppm): 5
HC Ratio: unlimited
Max Isotopes: all
MSn Iso RI (%): 75.00

DBE Range: not fixed
Apply N Rule: no
Isotope RI (%): 1.00
MSn Logic Mode: OR

Electron Ions: both
Use MSn Info: yes
Isotope Res: 10000
Max Results: 30

Event#: 2 MS(E-) Ret. Time : 0.373 -> 0.480 Scan# : 58 -> 74

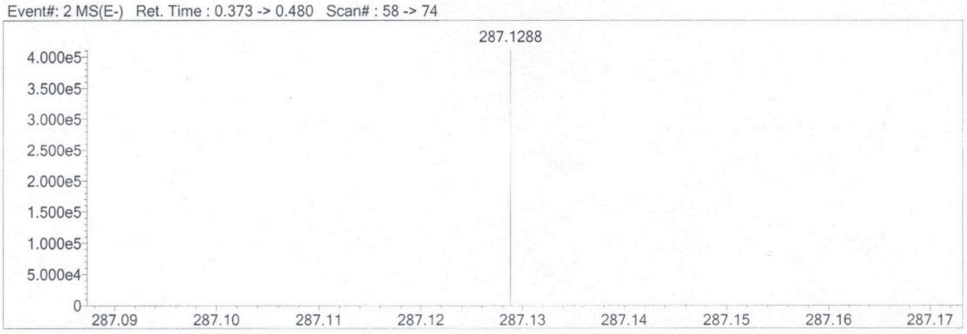

Formula (M)	Ion	Meas. m/z	Pred. m/z	Df. (mDa)	Df. (ppm)	DBE
C17 H20 O4	[M-H]-	287.1288	287.1289	-0.1	-0.35	8.0

Figure S1.1. (-)-HRESIMS of compound **1**.

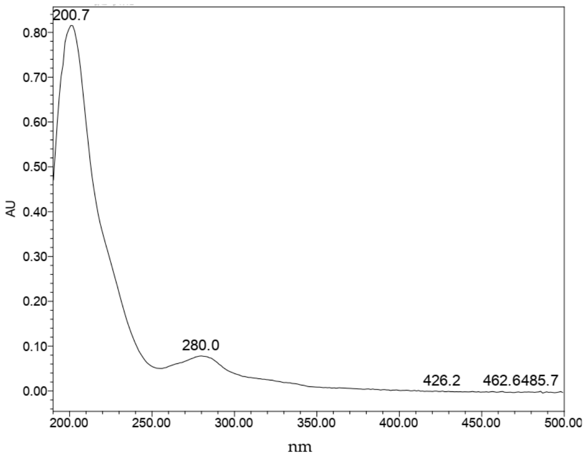

Figure S1.2. UV spectra of compound **1**.

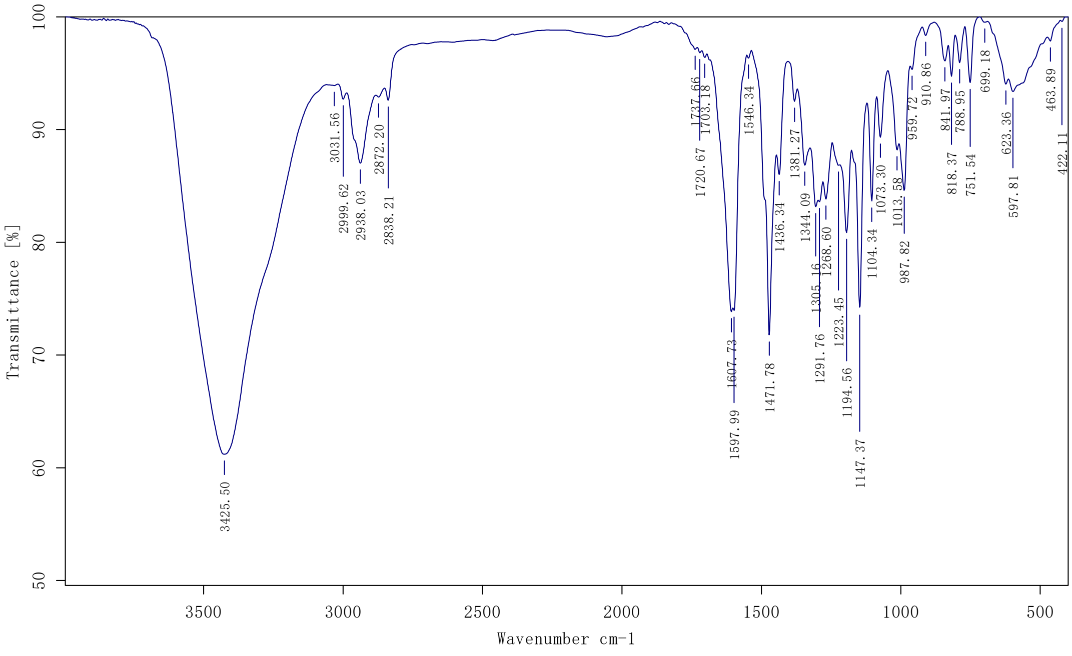

Figure S1.3. IR spectra of compound **1**.

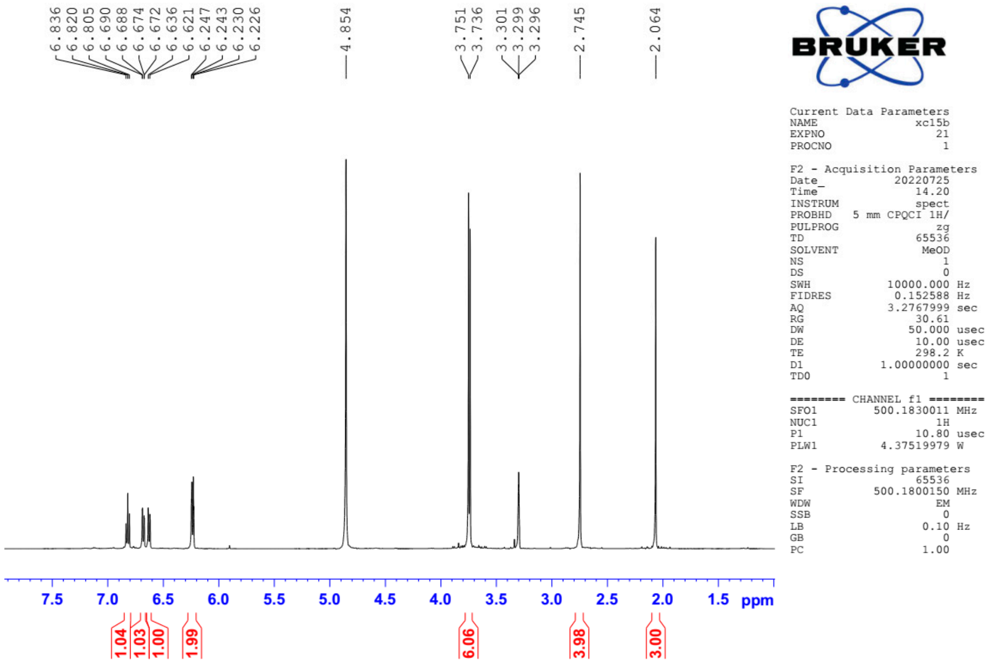

Figure S1.4. ^{1}H NMR (500 MHz, CD_3OD) spectrum of compound **1**.

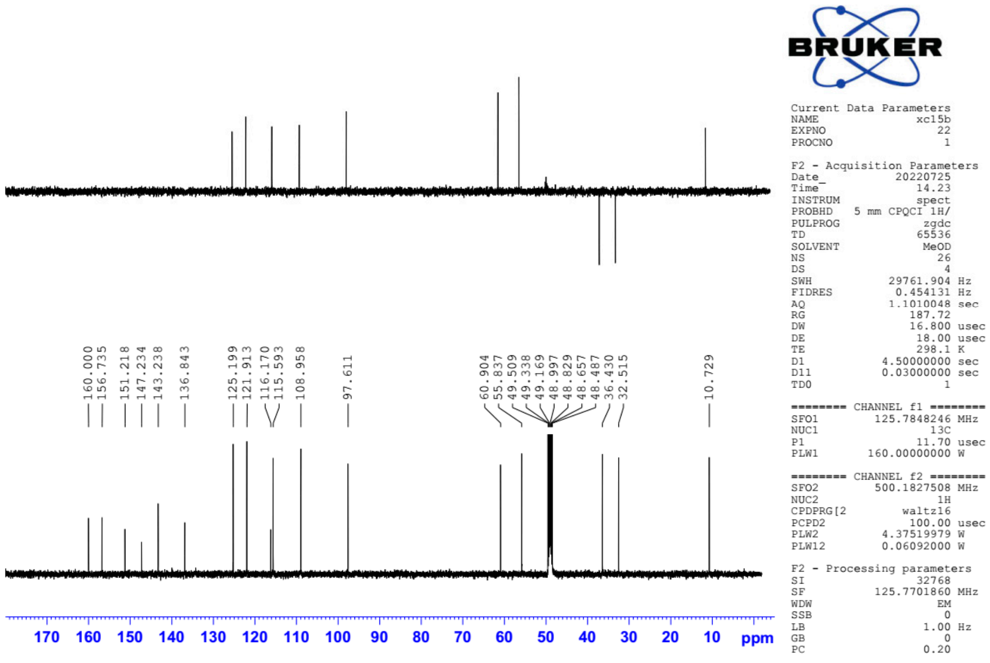

Figure S1.5. ^{13}C NMR and DEPT (125MHz, CD_3OD) spectrum of compound **1**.

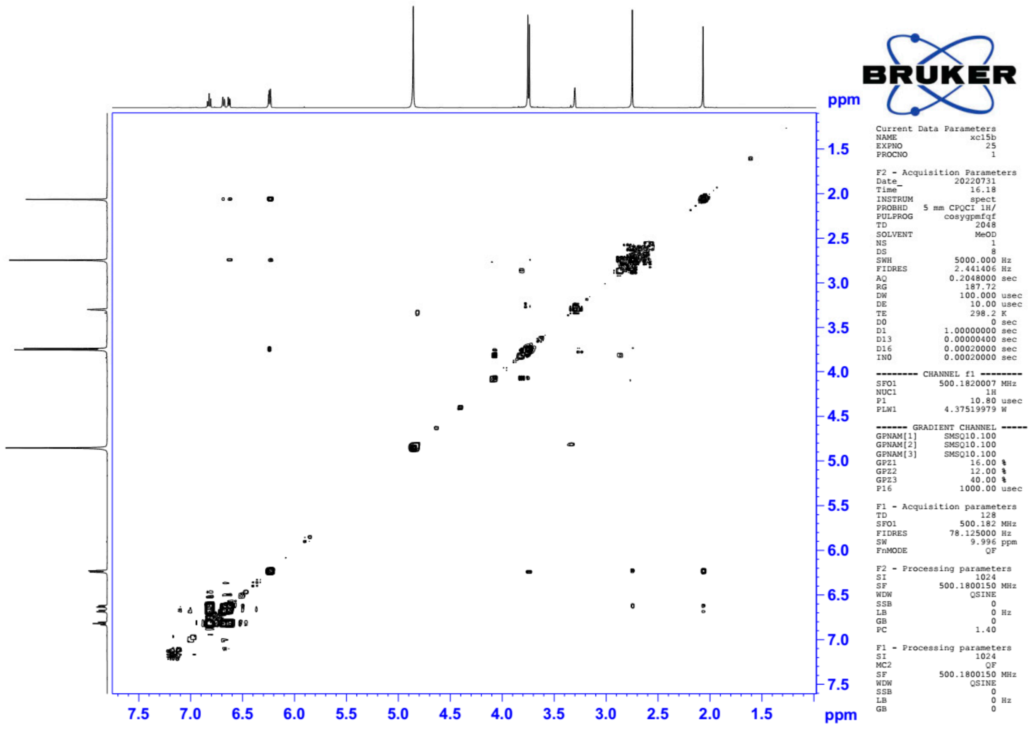

Figure S1.6. ^{1}H-^{1}H COSY spectrum of compound **1**.

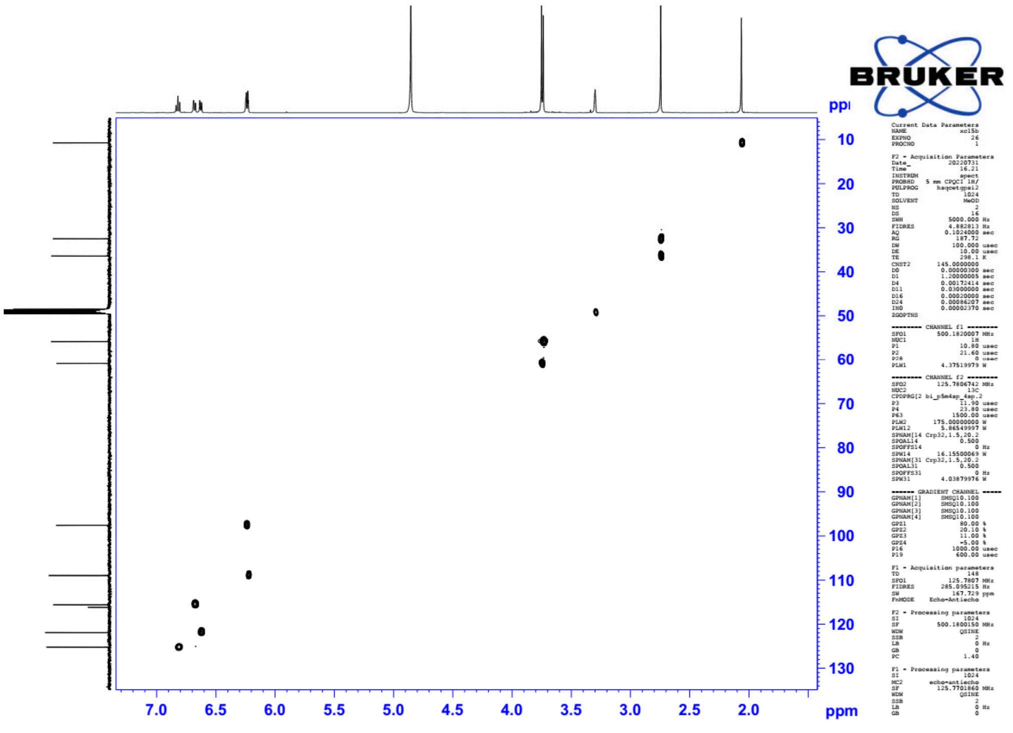

Figure S1.7. HSQC spectrum of compound **1**.

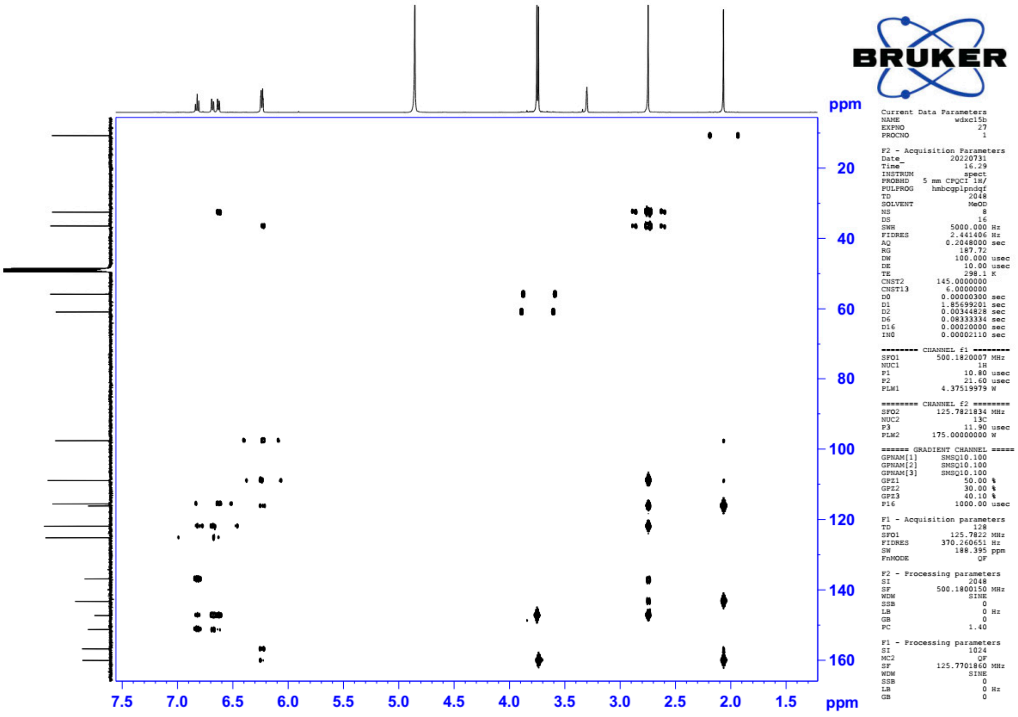

Figure S1.8. HMBC spectrum of compound **1**.

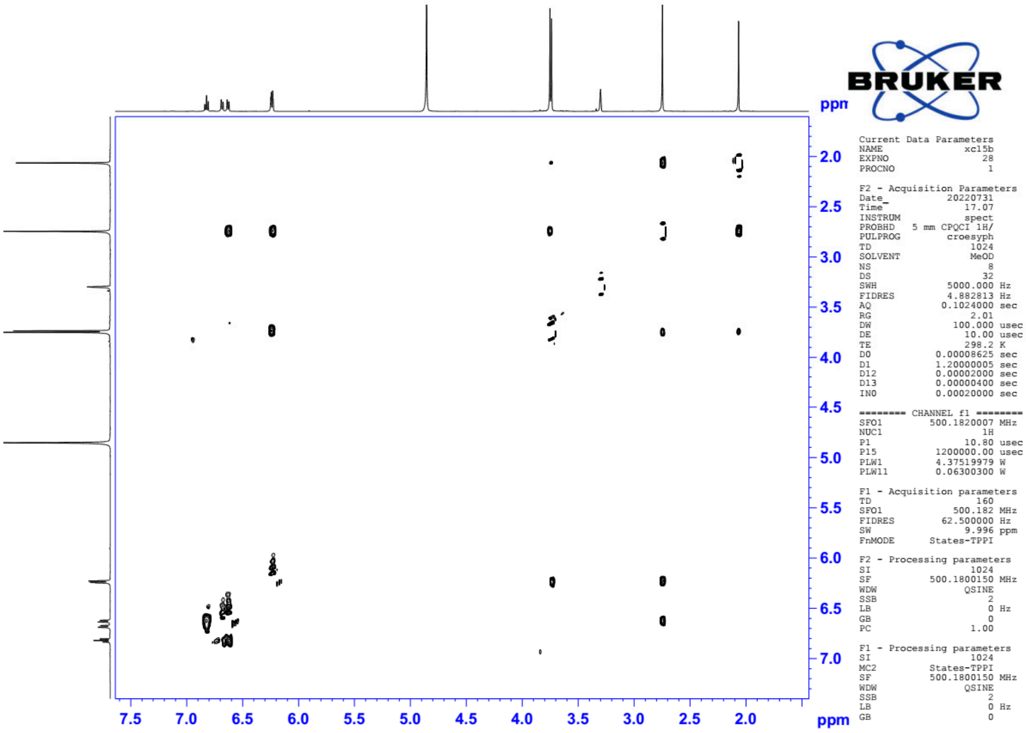

Figure S1.9. ROESY spectrum of compound **1**.

Error Margin (ppm):	5	DBE Range:	not fixed	Electron Ions:	both
HC Ratio:	unlimited	Apply N Rule:	no	Use MSn Info:	yes
Max Isotopes:	all	Isotope RI (%):	1.00	Isotope Res:	10000
MSn Iso RI (%):	75.00	MSn Logic Mode:	OR	Max Results:	30

Event#: 2 MS(E-) Ret. Time : 0.400 Scan# : 62

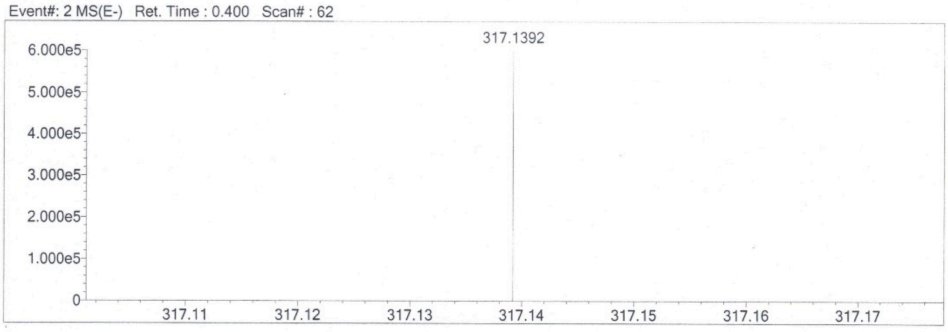

Formula (M)	Ion	Meas. m/z	Pred. m/z	Df. (mDa)	Df. (ppm)	DBE
C18 H22 O5	[M-H]-	317.1392	317.1394	-0.2	-0.63	8.0

Figure S2.1. (-)-HRESIMS of compound **2**.

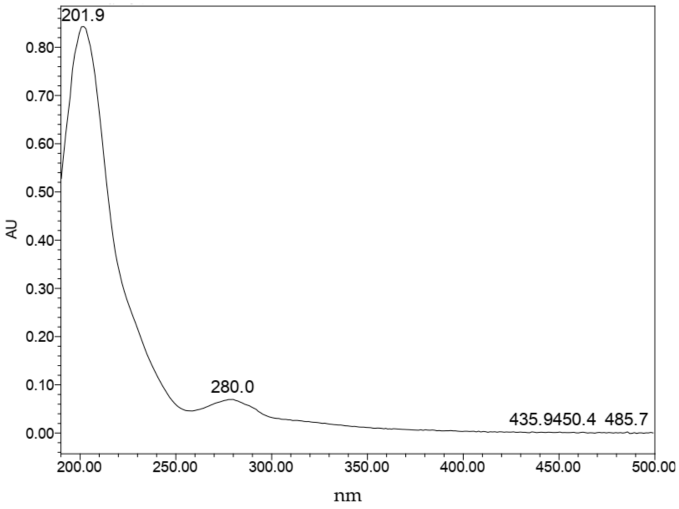

Figure S2.2. UV of compound **2**.

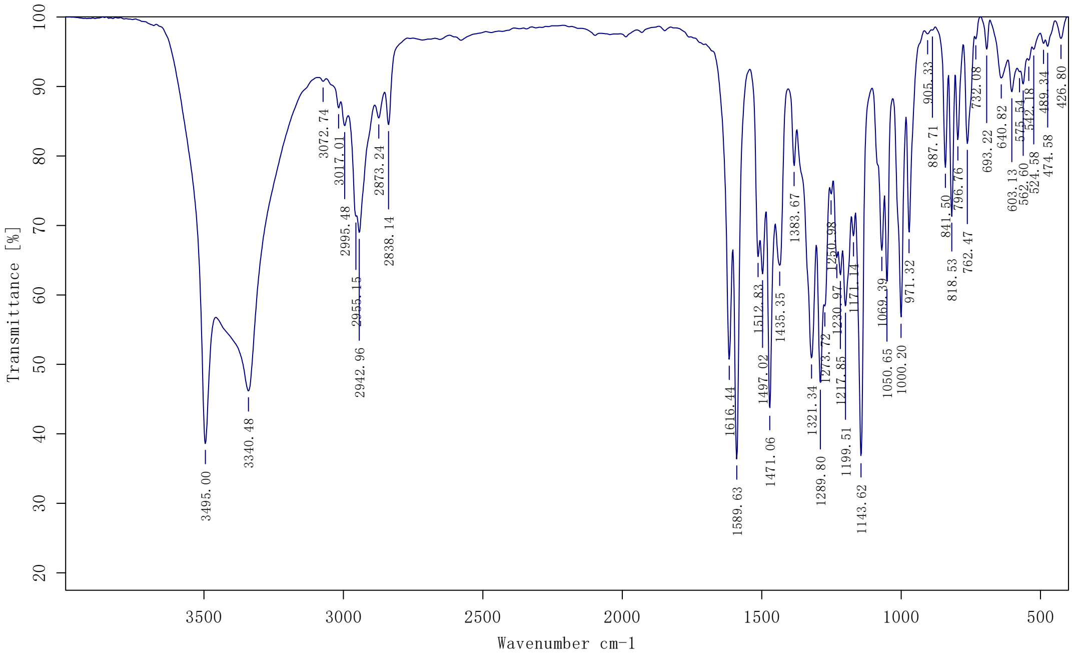

Figure S2.3. IR of compound **2**.

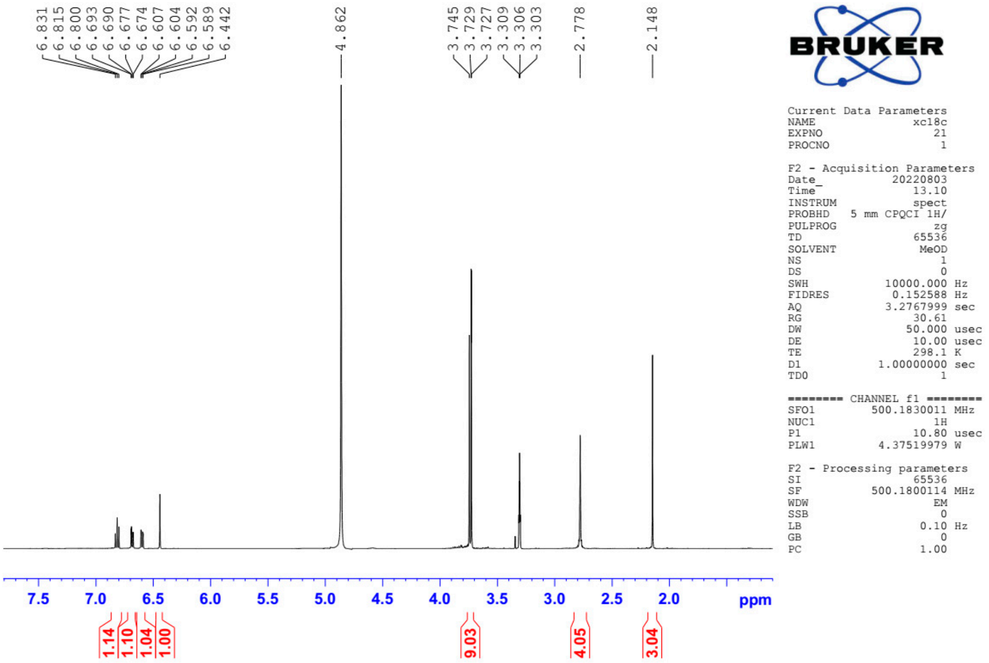

Figure S2.4. ^{1}H NMR (500 MHz, CD_3OD) spectrum of compound **2**.

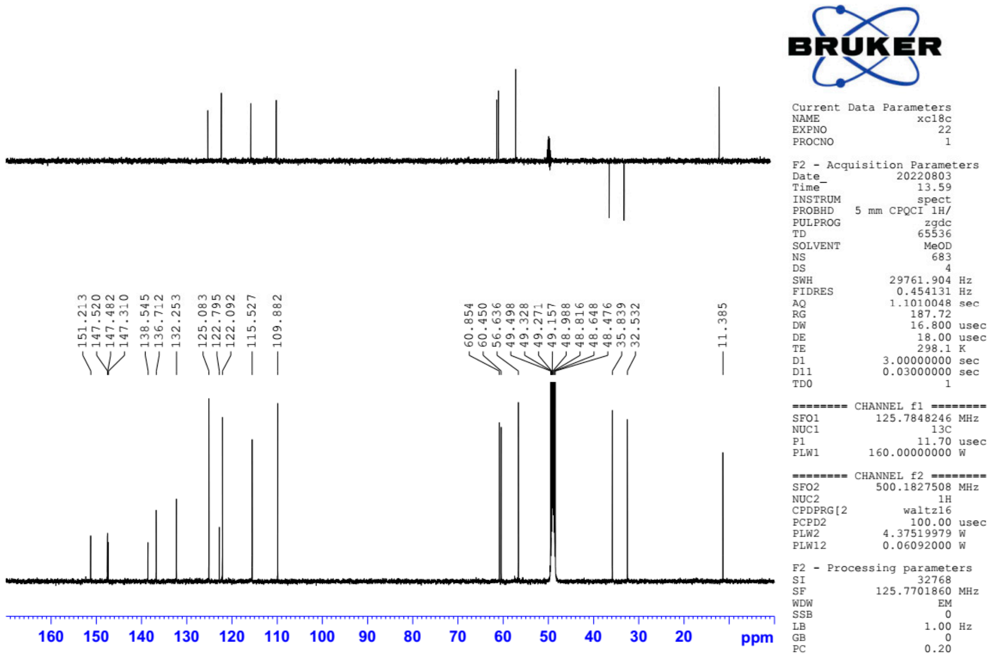

Figure S2.5. ^{13}C NMR and DEPT (125 MHz, CD_3OD) spectrum of compound **2**.

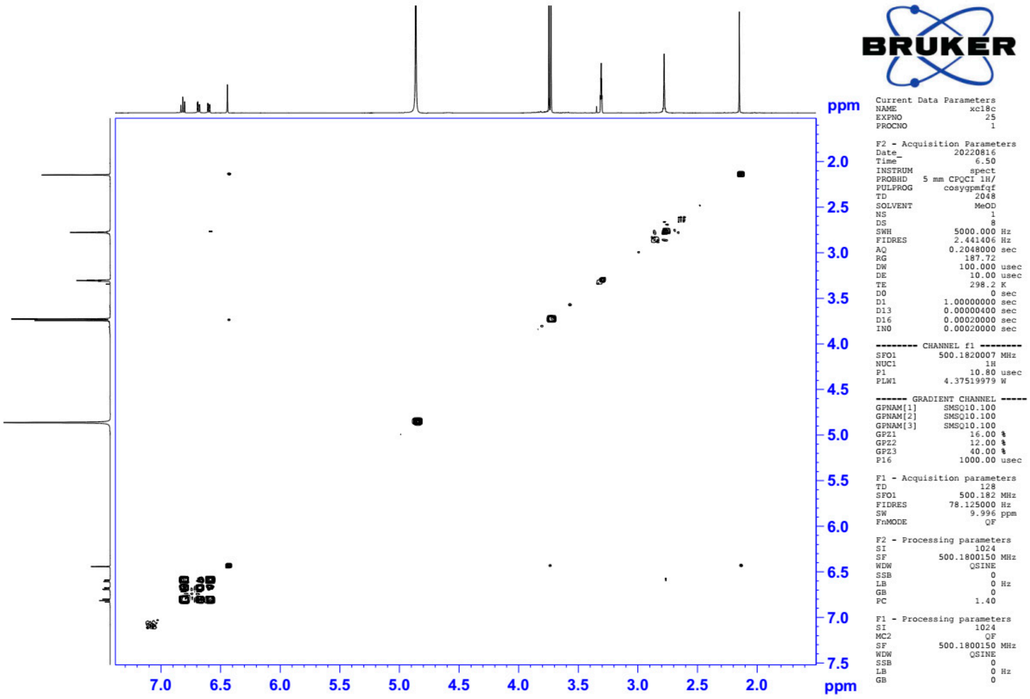

Figure S2.6. ^{1}H-^{1}H COSY spectrum of compound **2**.

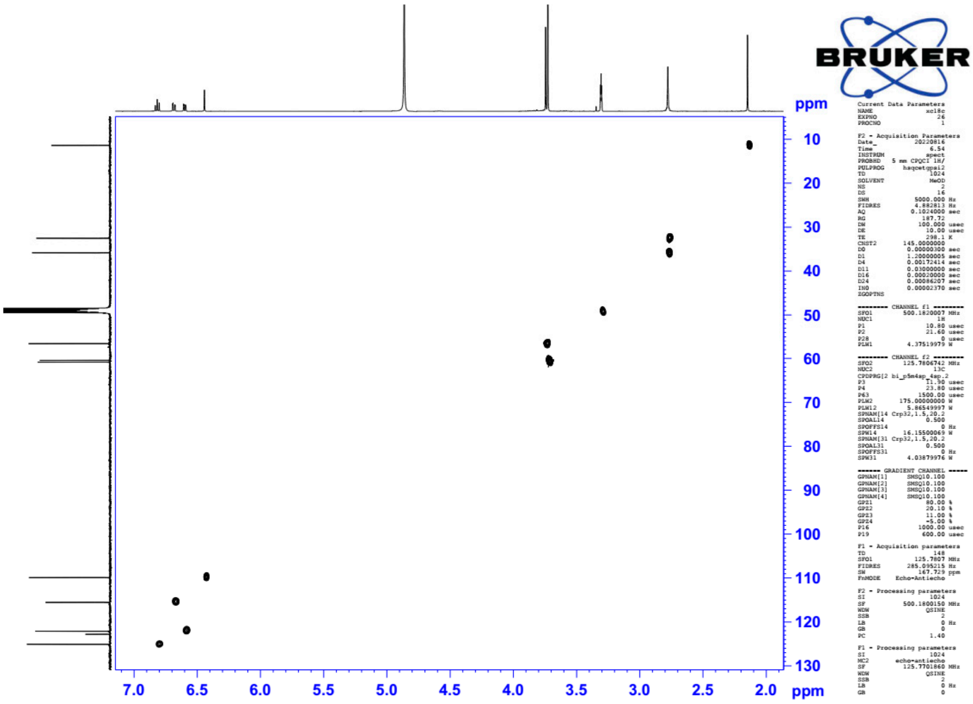

Figure S2.7. HSQC spectrum of compound **2**.

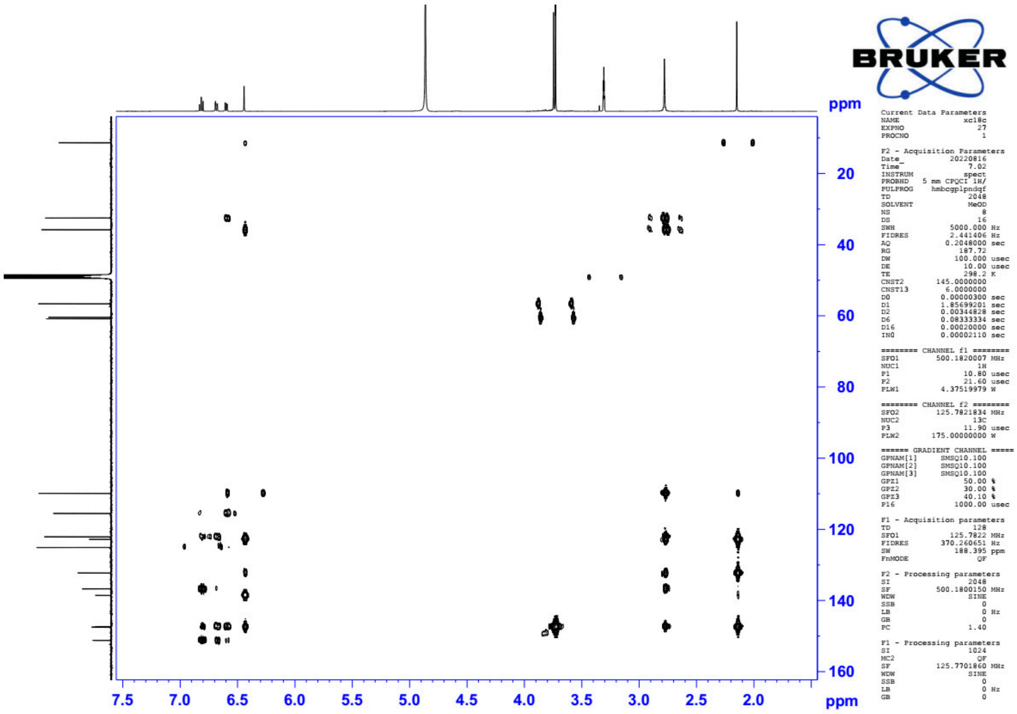

Figure S2.8. HMBC spectrum of compound **2**.

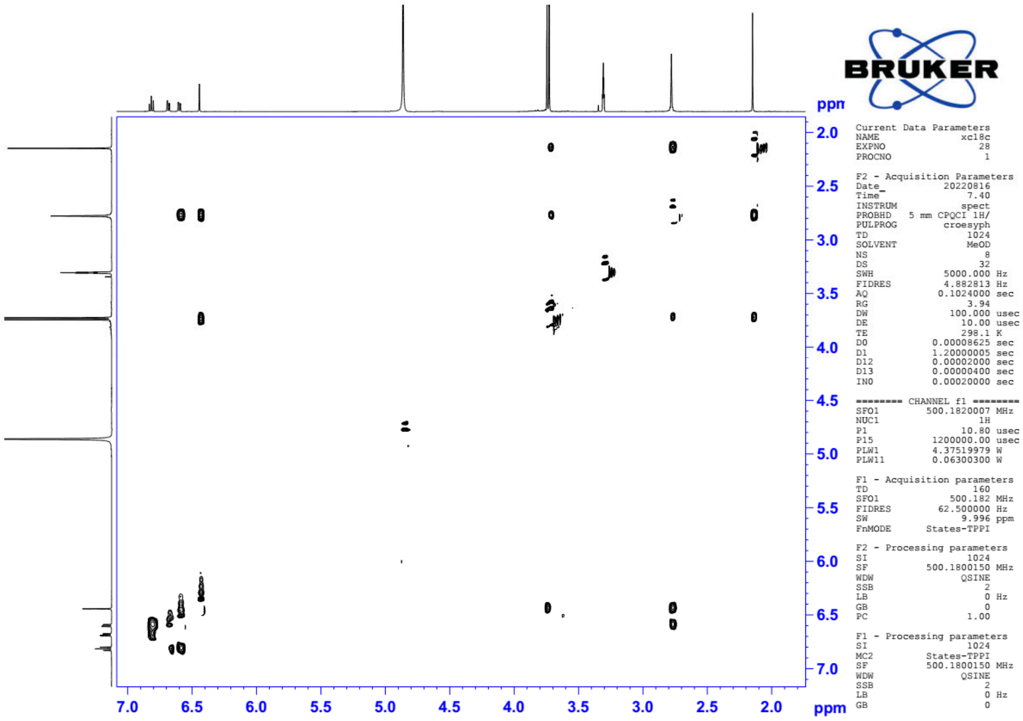

Figure S2.9. ROESY spectrum of compound **2**.

Error Margin (ppm):	5	DBE Range:	not fixed	Electron Ions:	both
HC Ratio:	unlimited	Apply N Rule:	no	Use MSn Info:	yes
Max Isotopes:	all	Isotope RI (%):	1.00	Isotope Res:	10000
MSn Iso RI (%):	75.00	MSn Logic Mode:	OR	Max Results:	30

Event#: 2 MS(E-) Ret. Time : 0.373 -> 0.773 Scan# : 58 -> 118

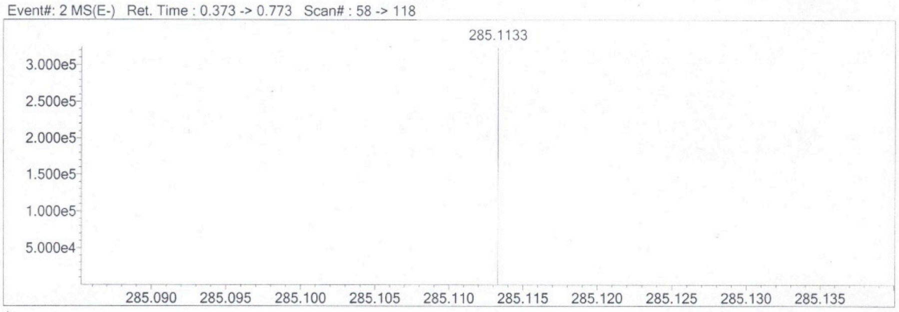

Formula (M)	Ion	Meas. m/z	Pred. m/z	Df. (mDa)	Df. (ppm)	DBE
C17 H18 O4	[M-H]-	285.1133	285.1132	0.1	0.35	9.0

Figure S3.1. (-)-HRESIMS of compound **3**.

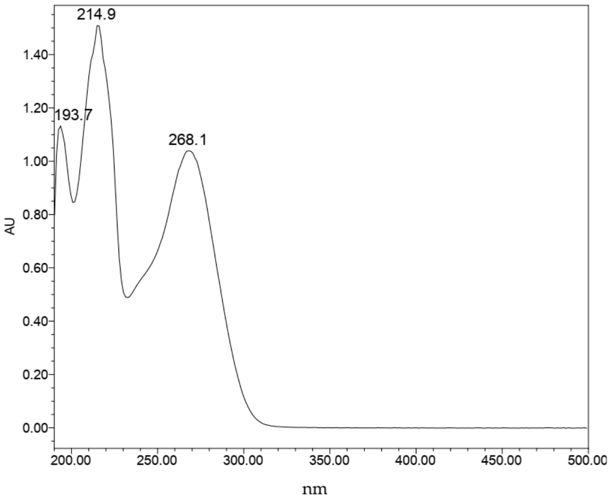

Figure S3.2. UV spectra of compound **3**.

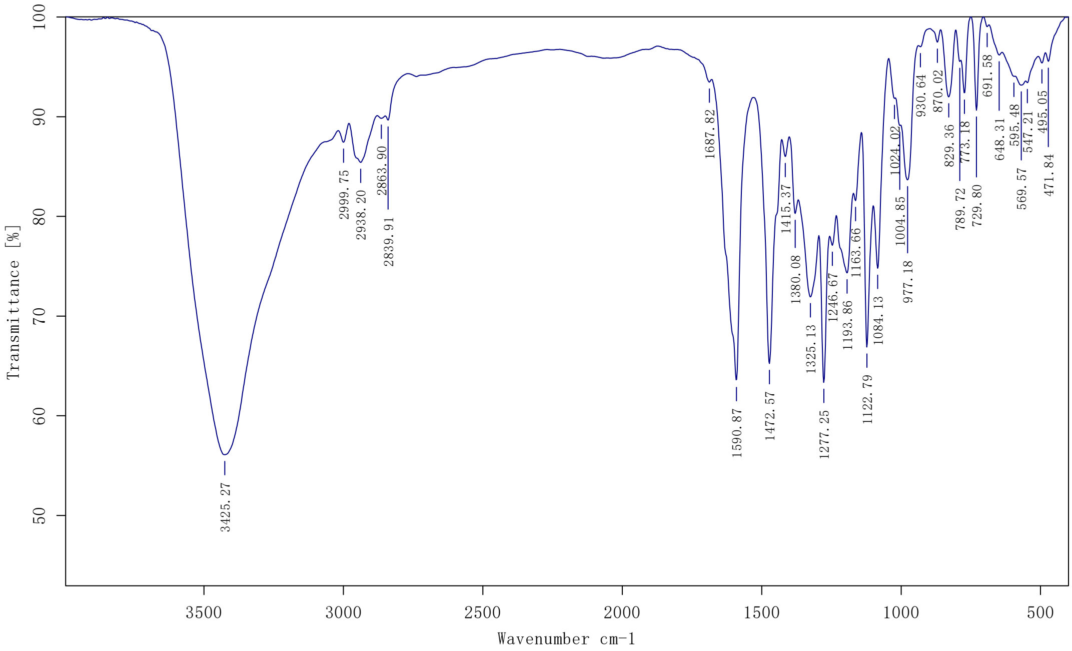

Figure S3.3. IR spectra of compound **3**.

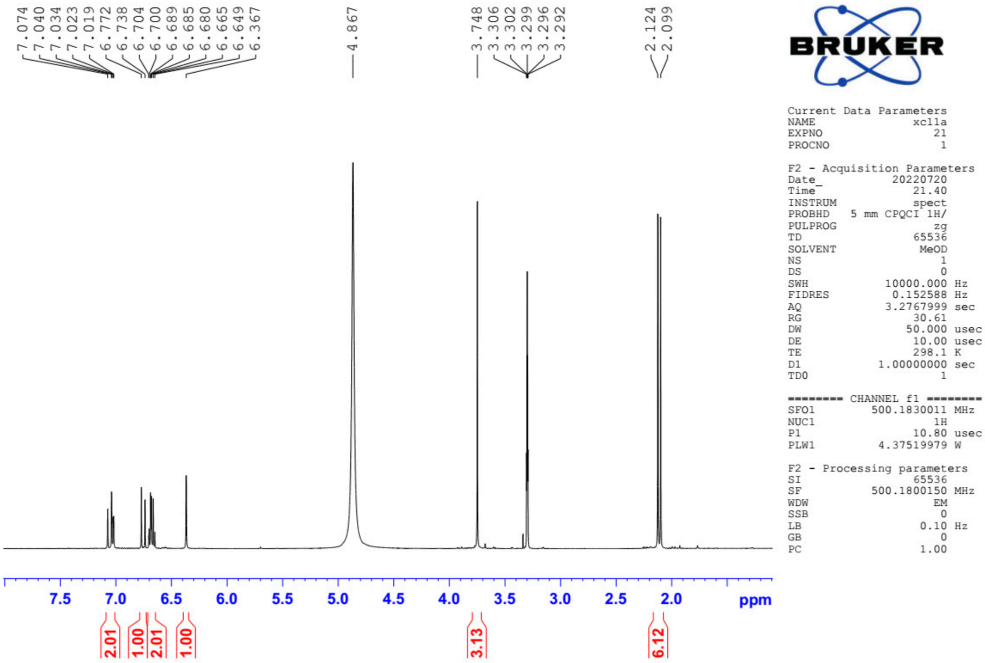

Figure S3.4. ^{1}H NMR (500 MHz, CD_3OD) spectrum of compound **3**.

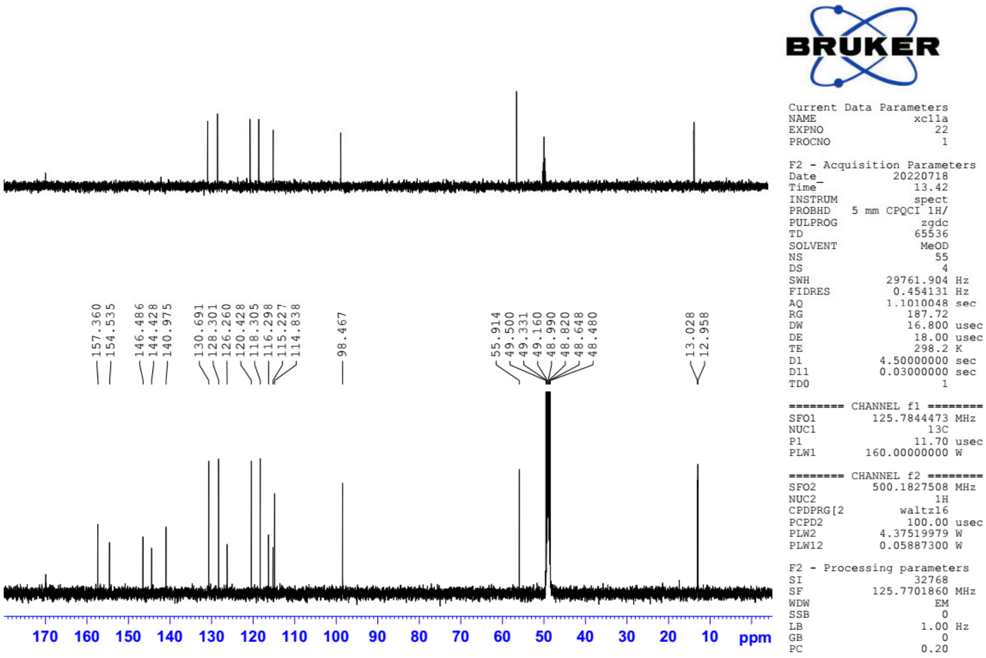

Figure S3.5. ^{13}C NMR and DEPT (125 MHz, CD_3OD) spectrum of compound **3**.

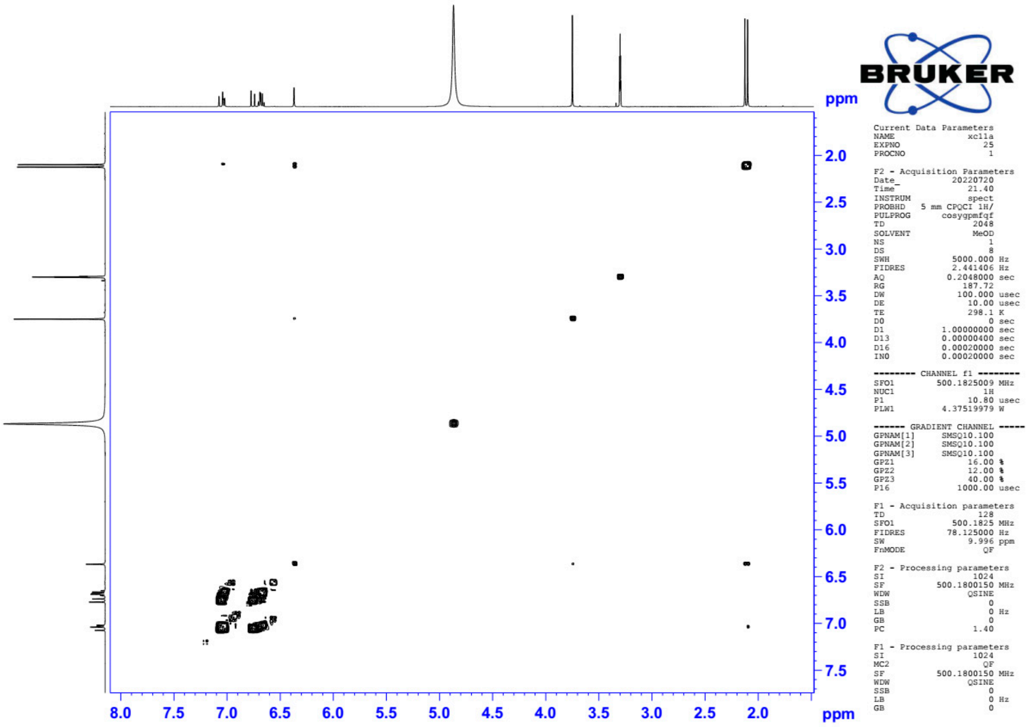

Figure S3.6. ^{1}H-^{1}H COSY spectrum of compound **3**.

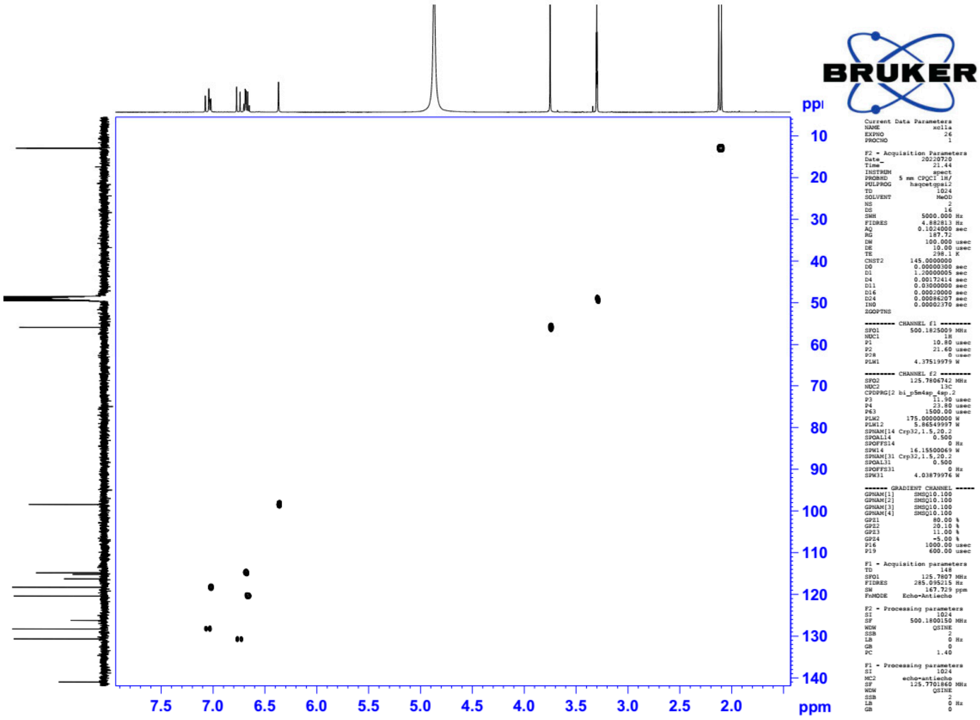

Figure S3.7. HSQC spectrum of compound **3**.

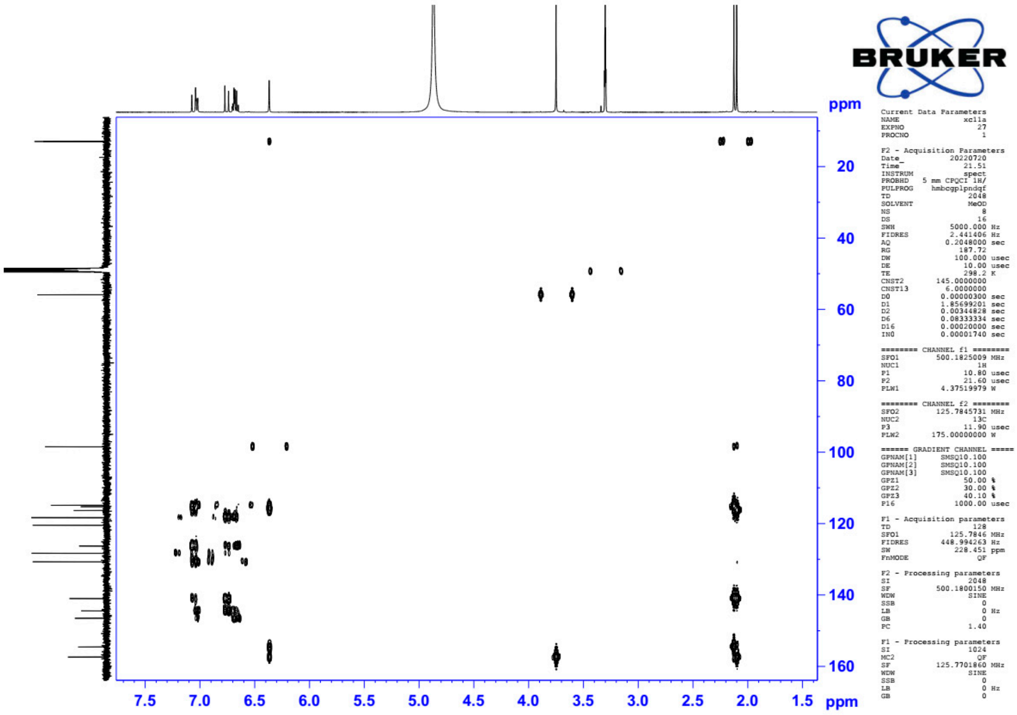

Figure S3.8. HMBC spectrum of compound **3**.

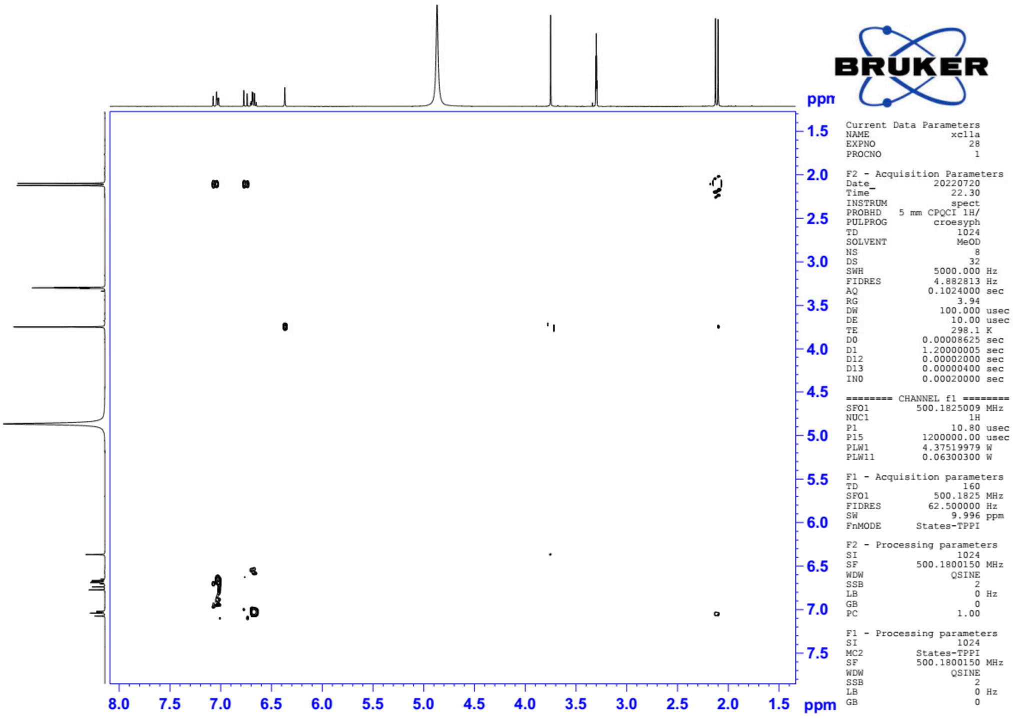

Figure S3.9. ROESY spectrum of compound **3**.
